# Supplementary material for: Immunogenicity of Hepatitis B Vaccination in Patients with Ulcerative Colitis on Infliximab Is Attenuated Compared to Those on 5-Aminosalicylic Acid Therapies: A Prospective Observational Study
Source: Vaccines (Basel). 2024 Mar 27;12(4):364. doi: 10.3390/vaccines12040364 (PMC11053706; doi:10.3390/vaccines12040364)
Supplement: Supplementary file 1 [file vaccines-12-00364-s001.zip › Supplementary File S2 .pdf]

Supplementary File S2: Baseline and clinical characteristics comparison after classifying the study population based on HBV vaccine response.

	**Adequate response (≥ 10-99, n=65)**	**Effective response (≥ 100 n=53)**	**p-value**
**Age** (Mean ± S.D)	34.1 ± (12.1)	34.7 ± (12.5)	0.806
**BMI** (Mean ± S.D)	26.2 ± (5.4)	24.7 ± (4.5)	0.093
**Gender, N (%)**			0.765
Male	35.0 (53.8%)	30.0 (56.6%)	
Female	30.0 (46.2%)	23.0 (43.4%)	
**Smoking, N (%)**			0.638
Smoker	9.0 (13.8%)	9.0 (17.0%)	
Non-Smoker	56.0 (86.2%)	44.0 (83.0%)	
**UC Type, N (%)**			0.762
E1	9.0 (13.8%)	5.0 (9.4%)	
E2	22.0 (33.8%)	19.0 (35.8%)	
E3	34.0 (52.3%)	29.0 (54.7%)	
**Inflmmatory markers** (Mean ± S.D)			0.373
Albumin, g/L	49 ± (6.4)	43 ± (5.8)	
CRP, mg/L	6.0 ± (4.5)	5.8 ± (4.4)	
Stool fecal calprotectin, ug/g	113 ± (12.0)	111 ± (11.7)	
