# Supplementary material for: Immunogenicity of Hepatitis B Vaccination in Patients with Ulcerative Colitis on Infliximab Is Attenuated Compared to Those on 5-Aminosalicylic Acid Therapies: A Prospective Observational Study
Source: Vaccines (Basel). 2024 Mar 27;12(4):364. doi: 10.3390/vaccines12040364 (PMC11053706; doi:10.3390/vaccines12040364)
Supplement: Supplementary file 1 [file vaccines-12-00364-s001.zip › Supplementary Table S1.pdf]

Supplementary Table S1: HBsAb levels in response to HBV vaccination.

HBsAb level group	Control group N (%)	Study group N (%)	Odds Ratio (95% CI)	p-value
10-99 IU/L	14.0 (23.3%)	51.0 (87.9%)	23.9 (8-64)	**<0.005**
≥100 IU/L	46.0 (76.7%)	7.0 (12.1%)		
